# The diagnostic potential of plasma SCUBE‐1 concentration for pulmonary embolism: A pilot study

**DOI:** 10.1111/crj.13588

**Published:** 2023-02-07

**Authors:** Lu Xiao, Minlian Wang, Sicong Yang, Shulin Li, Qijun Huang, Lan Xu, Yazhen Li, Yingyun Fu

**Affiliations:** ^1^ Shenzhen Institute of Respiratory Diseases The Second Clinical Medical College of Jinan University, Shenzhen People' s Hospital Shenzhen China; ^2^ Department of Metabolic and Bariatric Surgery The First Affiliated Hospital of Jinan University Guangzhou China; ^3^ Department of Cardiology The seventh Affiliated Hospital of Sun Yat sen University (Shenzhen) Shenzhen China

**Keywords:** CT pulmonary angiogram, diagnosis, plasma SCUBE‐1, pulmonary embolism

## Abstract

**Introduction:**

This study aimed to investigate the potential application of plasma signal peptide‐complement C1r/C1s, Uegf and Bmp1‐epidermal growth factor domain‐containing protein 1 (SCUBE‐1) as a biomarker in the diagnosis of pulmonary embolism (PE).

**Methods:**

This cross‐sectional study enrolled 177 patients who underwent PE diagnostic test and 87 healthy controls. The results of CT pulmonary angiogram (CTPA) were used as reference standards for PE diagnosis. The levels of SCUBE‐1 and D‐dimer in participants' plasma were detected with enzyme‐linked immunosorbent assay and compared among patients with confirmed PE, suspicious PE and healthy controls. The diagnostic values were analysed using receiver operating characteristic (ROC) curve analysis. In addition, differences in plasma SCUBE‐1 levels were compared among patients with different risk stratifications.

**Results:**

The plasma SCUBE‐1 concentration levels in patients with CTPA confirmed PE (14.28 ± 7.74 ng/ml) was significantly higher than those in the suspicious patients (11.11 ± 4.48 ng/ml) and in healthy control (4.40 ± 3.23 ng/ml) (*P* < 0.01). ROC curve analysis showed that at the cut‐off of 7.789 ng/ml, SCUBE‐1 has significant diagnostic value in differentiating PE patients from healthy control (AUC = 0.919, sensitivity = 81.25%, specificity = 92.13%), and the performance is more accurate than D‐dimer (cut‐off 273.4 ng/ml, AUC = 0.648, sensitivity = 65.75%, specificity = 67.42%). The combination of D‐dimer with SCUBE‐1 did not further improve the diagnostic value. However, SCUBE‐1 did not show significant diagnostic value in identifying PE among suspicious patients There was no significant difference in SCUBE‐1 level among different risk groups (*P* > 0.05).

**Conclusion:**

We believe that SCUBE‐1 could be a potential coagulation‐related marker for the diagnosis of PE.

## Graphical abstract

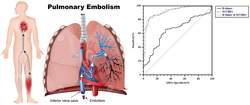

## 1 INTRODUCTION

Venous thromboembolism (VTE), which includes deep venous thrombosis (DVT) and pulmonary embolism (PE), is the third most common cardiovascular diagnosis following myocardial infarction and stroke.^1^ A domestic study on hospitalization and hospital mortality of VTE in China showed that the hospitalization rate of VTE increased from 3.2 in 2007 to 17.5/100 000 in 2016.^2^ In Europe, PE alone is about 296 000 cases, and fatal VTE is about 370 000 cases every year, in which rapid deterioration and lack to timely diagnosis are the most important reasons for fatality. About 34% of cases were sudden fatal PE, with only about 7% of fatal cases diagnosed before death. Early diagnosis is complicated by non‐specific clinical presentations, and PE remains one of the most commonly under diagnosed conditions.^3^, ^4^ Currently, the diagnosis of PE is primarily based on the amalgamation of blood tests with imaging analyses. Unfortunately, the D‐dimer test is only good for “ruling out” but not “ruling in” acute PE with high false‐positive rate. Computed tomography pulmonary angiography (CTPA), although reported as the ‘gold standard’, is inconvenient for regular clinical screen, and the usage is limited in patients with renal insufficiency and iodine‐containing contrast agent hypersensitivity.^5^, ^6^ Therefore, other non‐invasive, easy‐to‐detect and reliable biomarkers for PE diagnosis is still in need.

SCUBE1 [Signal peptide, CUB (complement C1r/C1s, Uegf, and Bmp1‐epidermal growth factor)] domain and EGF‐like domain containing is a cell surface protein that is expressed during early embryogenesis. The protein is mainly expressed in vascular endothelial cells (EC) and inactivated platelets and may play an important role in vascular biology.^7^, ^8^, ^9^, ^10^, ^11^, ^12^ It has been reported that SCUBE‐1 produced in megakaryocytes/platelets contributed to artery thrombosis in the absence of EC‐derived SCUBE‐1 and the its adhesive EGF‐like repeats are essential for crosslinking and stabilizing platelet aggregates during thrombus formation in vivo.^13^, ^14^ The involvement of SCUBE‐1 in thrombosis may indicate its potential for identification of thrombosis‐related clinical condition risks. Therefore, this study aimed to explore the potential diagnostic value of SCUBE‐1 for PE.

## 2 MATERIALS AND METHODS

### 2.1 Study design and population

In a cross‐sectional study setting, patients aged >18 years who underwent CTPA diagnosis due to suspicion of PE between December 2016 and December2018 in the Second Clinical Medical School affiliated to Jinan University were included. Patients were excluded if they had acute coronary syndrome (ACS), acute ischaemic stroke (AIS) and other ischemic diseases such as mesenteric, liver/renal/heart failure, congenital cardiomyopathy, severe valvular heart disease, haematological disorder or rheumatologic disease. Healthy individuals were recruited from the Department of Physical Examination. The plasma SCUBE1 levels in confirmed PE patients, suspicious but negative patients and healthy individuals were compared. The diagnostic values of SCUBE1 to discriminate different populations were explored.

The study protocol was in accordance with the ethics guidelines of the Declaration of Helsinki and approval was obtained from the ethical committee of the Second Clinical Medical School affiliated to Jinan University (Shenzhen People's Hospital). Written informed consent was obtained from all the participants before enrolment.

### 2.2 Specimen collection and measurements

A 6‐ml venous blood sample was collected from all the participants from the brachial vein into EDTA anticoagulant tube. The Samples were centrifuged at 1000 *g*, 4°C for 15 min, and the plasma was stored at −80°C before further experiment. Regular blood biochemical tests were performed using an autoanalyzer (Thermo Fisher Scientific 1510, Shanghai, China). Levels of SCUBE‐1 and D‐dimer in the plasma were detected using commercial enzyme linked immunosorbent assays (Cusabio, Cat No: CSB‐E15005h, Wuhan, China), respectively.

### 2.3 Other data collection

Data related to demographic and clinical characteristics, including age, gender, smoking status, comorbidities and disease histories such as hypertension, coronary atherosclerotic disease (CAD), stroke, chronic obstructive pulmonary disease (COPD) diabetes and tumours were documented.

### 2.4 Statistical analysis

All statistical analyses were performed using the SPSS Statistics software (Version 22.0, IBM, Armonk, NY, USA) and GraphPad Prism (Version 6.02, GraphPad Software, San Diego, CA, USA). Normally distributed quantitative parameters were expressed as mean ± standard deviation. Student's *t*‐test was used to compare the continuous variables with normal distribution between the two groups. Non‐normally distributed parameters were expressed as median and range and compared among groups using Kruskal–Wallis H test. The categorical variables were presented as frequency and percentage with differences between groups compared with chi‐squared test. Receiver operating characteristic (ROC) curve analysis was used to detect the sensitivity, specificity and predictive value of SCUBE‐1 for detection of PE against health individual or suspicious but negative patients. Paired *t*‐test was conducted to compare the SCUBE1 at the time of inclusion and the end of follow‐up. Statistical significance was determined by a *P*‐value < 0.05.

## 3 RESULTS

### 3.1 Patient characteristics

Total 177 patients who underwent CTPA test and met the eligible criteria were included. Among them there were 143 confirmed PE diagnosis (PE group) and 34 negative CTPA test results (suspicious PE). In addition, 89 healthy control participants were recruited. The demographic and clinical features are presented in Table 1. The comparison results showed that suspicious PE patients (mean age 62.12 ± 14.21) were significantly (*P* < 0.05) older than healthy controls (mean age 53.97 ± 13.74) and contained little male individuals (18% vs. 41%, *P* < 0.05). No significant differences were detected between PE and suspicious PE patients.

**TABLE 1:** Characteristics of individuals enrolled in the study

Variables	PE(*n* = 143)	Suspicious PE(*n* = 34)	Healthy controls(*n* = 89)	*P*
Age, years	58.04 ± 16.73	62.12 ± 14.21	53.97 ± 13.74	0.022*
Men (%)	77(53.85)	18(52.94)	41(46.07)	0.501
Smoking, *n* (%)	40(27.97)	7(20.59)	‐	0.370
Baseline diseases				
Hypertension, *n* (%)	40(27.97)	15(44.12)	‐	0.068
CHD, *n* (%)	18(12.59)	8(23.53)	‐	0.113
Stroke, *n* (%)	14(9.79)	4(11.77)	‐	0.754
COPD, *n* (%)	7(4.90)	5(14.71)	‐	0.056
Diabetes, *n* (%)	14(9.79)	5(14.71)	‐	0.371
Tumor, *n* (%)	16(11.19)	0(0.00)	‐	0.088
Signs and symptoms				
Chest pain, *n* (%)	30(20.98)	8(23.53)	‐	0.817
Dyspnoea, chest tightness, *n* (%)	81(56.64)	15(44.12)	‐	0.084
Haemoptysis, *n* (%)	18(12.59)	2(5.88)	‐	0.373
Syncope, *n* (%)	12(8.39)	0(0.00)	‐	0.126
Risk stratification				
Low risk	69(48.25)	‐	‐	‐
Low‐intermediate risk	45(31.46)	‐	‐	‐
High‐intermediate risk	17(11.89)	‐	‐	‐
High risk	12(8.39)	‐	‐	‐

Abbreviations: CHD, coronary atherosclerotic heart disease; COPD, chronic obstructive pulmonary disease; PE, pulmonary embolism; PE, pulmonary embolism.

* *P* < 0.05 compared with control subjects.

### 3.2 Diagnostic value of SCUBE‐1 for PE

**FIGURE 1:**
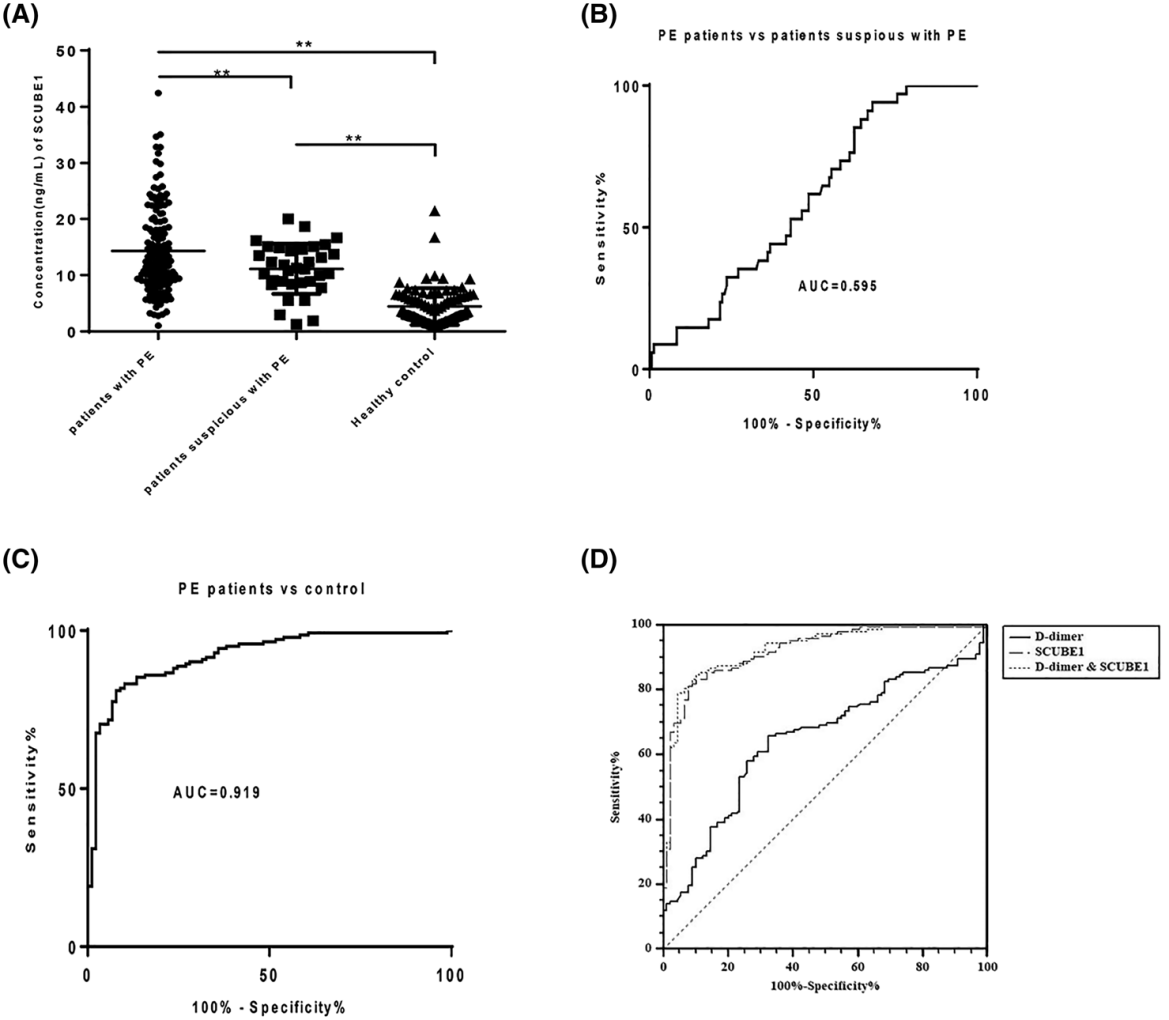
(A) Scatter plot of SCUBE‐1 levels in patients with PE versus patients suspicious with PE and healthy controls. (B) ROC curve of SCUBE‐1 in differentiating CTPA confirmed and suspicious but negative PE patients. (C) The ROC curve of SCUBE‐1 in the differentiating patients with PE from healthy controls. (D) Comparisons between ROC curves of SCUBE‐1, D‐dimer and the combination of both markers

The plasma SCUBE‐1 concentration among PE patients (14.28 ± 7.74 ng/ml), suspicious PE patients (11.11 ± 4.48 ng/ml) and healthy controls (4.40 ± 3.23 ng/ml) were significantly different. As shown in Figure 1A, the plasma SCUBE‐1 level in the patients with PE was higher than those in suspicious PE and healthy controls (*P* < 0.01), and the level of SCUBE‐1 in suspicious PE patients was also higher than that in healthy controls (*P* < 0.01). The plasma D‐dimer concentration between PE patients (1275.89 ± 2785.27 ng/ml) and healthy controls (329.65 ± 399.23 ng/ml) were also different. The results of ROC analysis showed that the plasma SCUBE1 level did not efficiently distinguish PE patients from suspicious PE (AUC = 0.596, sensitivity = 31.91%) although a high specificity (94.12%) at the cut‐off of 16.635 ng/ml was detected (Figure 1B). On the other hand, when applied to discriminate PE from health control (Figure 1C), plasma SCUBE‐1 showed an area under the curve (AUC) of 0.919(95%, CI = 0.883–0.955). At the cut‐off of 7.789 ng/ml, a sensitivity of 81.25% and specificity of 92.13% were detected. The diagnostic value of SCUBE‐1 in distinguishing PE patients from healthy controls were significantly higher (*P* < 0.01) than D‐dimer (cut‐off 273.4 ng/ml, AUC = 0.648, sensitivity = 65.75%, specificity = 67.42%) (Figure 1D), and combination with D‐dimer did not further improve the diagnostic value of SCUBE‐1 (*P* > 0.05). According to the SCUBE1 and D‐dimer levels in the normal control group, the upper limit of the ‘normal range’ of the two groups was 10.86 and 1128.11 ng/ml, respectively. As shown in Tables 2A and 2B, the sensitivity, specificity, positive predictive and negative predictive value of SCUBE1 for diagnosing PE are 58.04%, 97.75%, 97.65% and 59.18% respectively. However, the above four values of D‐dimer are 20.17%, 94.38%, 82.76% and 41.38%. As shown in Tables 3A and 3B. The sensitivity difference between the two tests for diagnosing PE was statistically significant (χ^2^ = 41.93, *P* < 0.005). And there was no significant difference in the specificity of PE diagnosis (χ^2^ = 1.29, *P* > 0.05). Combined with the area under ROC curve, this result is further verified (Figure 1D).

**TABLE 2A:** Diagnostic evaluation of SCUBE‐1 for PE

	PE	Control	Total
SCUBE‐1(+)	83	2	85(97.65%)
SCUBE‐1(−)	60	87	147(59.18%)
Total	143(58.04%)	89(97.75%)	232

**TABLE 2B:** Diagnostic evaluation of D‐dimer for PE

	PE	Control	Total
D‐dimer(+)	24	5	29(82.76%)
D‐dimer(−)	119	84	203(41.38%)
Total	143(20.17%)	89(94.38%)	232

Abbreviation: PE, pulmonary embolism.

**TABLE 3A:** Results of SCUBE‐1 and D‐dimer test in 143 PE patients

	D‐dimer(+)	D‐dimer(−)
SCUBE‐1(+)	12	71
SCUBE‐1(−)	12	48

**TABLE 3B:** Results of SCUBE‐1 and D‐dimer test in 89 non‐PE patients

	D‐dimer(+)	D‐dimer(−)
SCUBE‐1(+)	0	2
SCUBE‐1(−)	5	82

Abbreviation: PE, pulmonary embolism.

**FIGURE 2:**
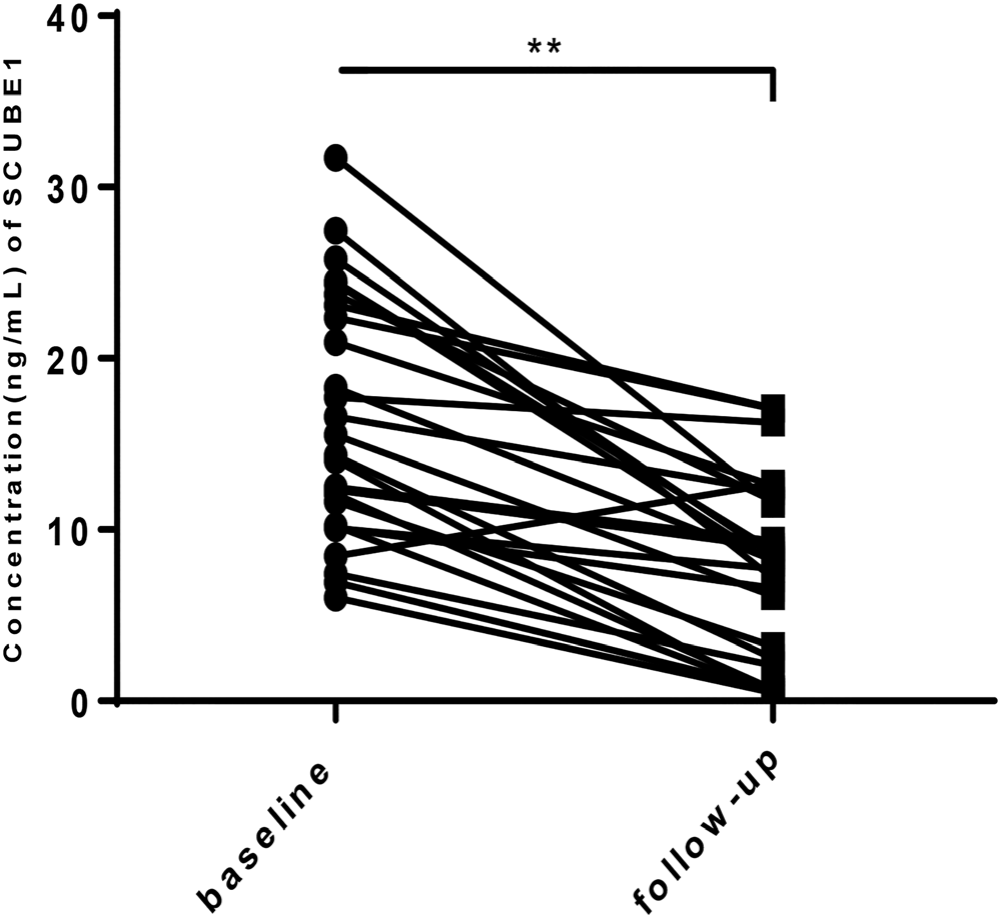
Changes of plasma concentration of SCUBE1 after 3‐month follow‐up

There were 26 of the PE patients who were followed up for 3 months after treatment, the CTPA was conducted at the end of follow‐up, and the results showed that the embolus of all the patients were smaller. Plasma SCUBE1 were also detected in 26 of the PE patients after 3‐month follow‐up. SCUBE1 concentration in the follow‐up samples was significantly lower (*P* < 0.05; Figure 2).

**FIGURE 3:**
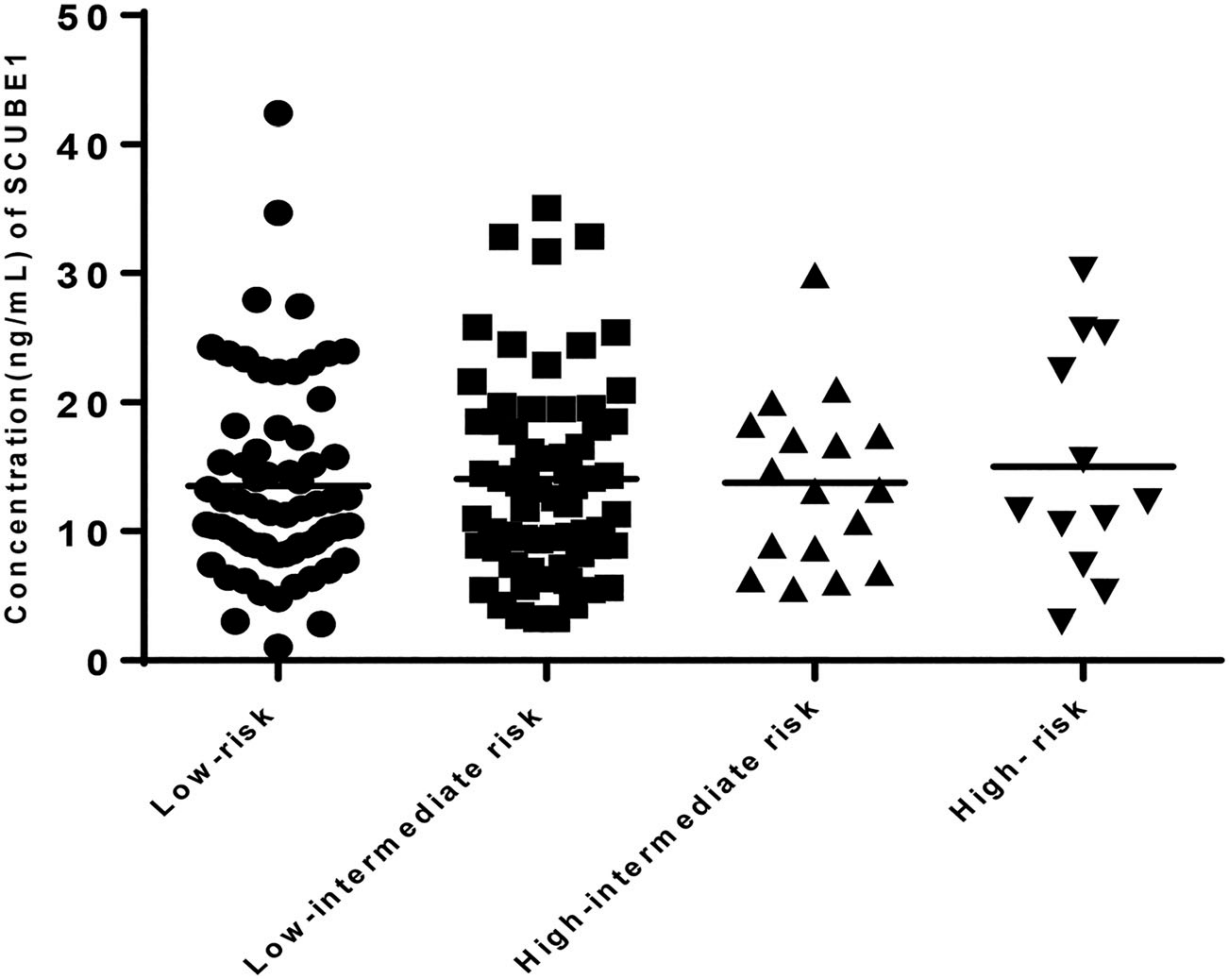
Scatter plot of SCUBE1 levels in patients of different risk stratifications

In order to explored the correlation of SCUBE‐1 in different risk groups, we divided the PE group into four subgroups according to their risk stratification based on the guideline of pulmonary embolism, 2018. No significant differences were detected in plasma SCUBE‐1 levels among those groups (Figure 3).

## 4 DISCUSSION

In this study, we aimed to investigated the early PE diagnostic value of SCUBE‐1, a novel platelet activation marker. We found that significantly higher serum SCUBE‐1 levels in patients with PE and suspicious with PE compared with healthy controls. The ROC analysis showed that AUC of SCUBE‐1 was larger than D‐dimer in differentiating patients with PE from healthy controls.

SCUBE‐1 has been investigated in various diseases, such as pulmonary hypertension,^15^ AIS^16^ and gestational diabetes mellitus (GDM). SCUBE‐1 was mainly expressed on human platelet surface, and platelets were activated to participate in platelet focusing, resulting in the formation of embolism.^17^, ^18^ It has been reported that SCUBE‐1 is a coagulation marker^19^, ^20^ associated with injury of vein endothelial cells and can be used as a biomarker for embolic diseases.^21^, ^22^, ^23^ In addition, mutation of SCUBE‐1 gene increases the risk of venous thrombosis.^24^ SCUBE‐1 has potential receptor, platelet endothelial aggregation receptor‐1 (PEAR‐1), and our previous studies have found that PEAR‐1 gene mutation is related to PE.^25^ These findings indicated SCUBE‐1 may be used as a potential coagulation‐related marker for the diagnosis of PE.

PE is an endpoint of thrombus formation. Previous study^23^ concluded SCUBE‐1 might rise from the early stage of PE. Similarly, this study showed that levels of SCUBE‐1 in suspicious PE patients were significantly higher than healthy controls. However, the results did not show that SCUBE‐1 could efficiently discriminate suspicious PE from the confirmed PE patients. Also, patients with different PE risk stratification did not show differences in plasma SCUBE‐1 levels. Therefore, the application value of SCUBE‐1 in clinical diagnosis and early warning of PE needs to be further confirmed.

D‐dimer is a product of the fibrinolysis process, and coagulation activation is recommended as an initial test in all PE guidelines. However, its clinical usage is limited due to high false‐negative rate.^26^, ^27^, ^28^, ^29^, ^30^, ^31^ The results of our study showed that SCUBE‐1 is less sensitive than D‐dimmer in the diagnosis of PE, but it has great advantages in specificity. Therefore, it may be considered as a first‐line screening tool to rule out the false PE risk. Unfortunately, no further improvement in diagnostic efficiency was achieved by combination of SCUBE‐1 and D‐dimer. Therefore, a reliable and simple way to confirm PE using plasma biomarkers still needs further exploration.

## 5 LIMITATIONS

It was undeniable that this study was a single‐centre study with small sample size. It is well known that in different studies, this was due to differences in race, sample size, design, environment and enrolment, Therefore, the interpretation of the results in this study is limited. We only initially explored the diagnostic value of SCUBE‐1. Therefore, larger and more diverse studies are needed in the future to verify the reliability of our conclusions. Moreover, the definition of risk stratification to PE patients may be further clarified to confirm values of markers in PE identification.

In summary, this study proved that the level of SCUBE‐1 was elevated in patients with PE. The results of this study support SCUBE‐1 as potential diagnostic biomarkers for PE.

## CONFLICT OF INTEREST

The authors declared that they have no conflict of interest.

## AUTHOR CONTRIBUTIONS

Xiao Lu was mainly responsible for clinical patient communication, specimen collection, experimental operation and article writing. Minlian Wang contributed the purchase of experimental instruments and consumables, part of the experimental operation and the revision of the paper. Sicong Yang collected specimens and discussed part of the opinion of the article. Shulin Li, Qijun Huang, Lan Xu and Yazhen Li contributed to clinical patient communication and specimen collection. Yingyun Fu communicated the article and revised the final draft.

## ETHICS STATEMENT

The study was designed and implemented in accordance with the Declaration of Helsinki, and the protocol was approved by the ethics committee of the Shenzhen People's Hospital. All patient data were anonymized before analysis.

## Data Availability

The data that support the findings of this study are available on request from the corresponding author. The data are not publicly available due to privacy or ethical restrictions.
